# Management of life‐threatening acute respiratory syndrome and severe pneumonia secondary to COVID‐19 in pregnancy: A case report and literature review

**DOI:** 10.1002/ccr3.3485

**Published:** 2020-11-11

**Authors:** Salwa Yaqoub, Shamsa Ahmad, Zaina Mansouri, Abdulrouf Pallivalapila, Wessam El Kassem, Muna Maslamani, Mahmoud Abu Jubara, Fathima Minisha, Asma Tarannum, Isaac Babarinsa, Ahmed Abdussalam, Hamdy Al Sayed, Teresa Rivero, Aftab Mohammad Azad, Binny Thomas, Moza Al Hail

**Affiliations:** ^1^ Obstetrics Women's Wellness and Research Center Hamad Medical Corporation Doha Qatar; ^2^ Obstetrics Emergency Women's Wellness and Research Center Hamad Medical Corporation Doha Qatar; ^3^ Pharmacy Executive Director's Office Hamad Medical Corporation Doha Qatar; ^4^ Infectious Disease Communicable Disease Center Hamad Medical Corporation Doha Qatar; ^5^ Hamad General Hospital Hamad Medical Corporation Doha Qatar; ^6^ Neonatal Intensive Care Unit Hamad Medical Corporation Doha Qatar; ^7^ Obstetrics Cuban Hospital Hamad Medical Corporation Doha Qatar

**Keywords:** convalescent plasma, COVID‐19, ECMO, maternal, pandemic, perinatal, pharmacological interventions

## Abstract

Evidence‐based treatment involving multidisciplinary decision making is warranted to treat COVID‐19 in pregnancy. This case presents the management of a critically ill pregnant women infected with SARS‐CoV‐2.

## Graphical abstract

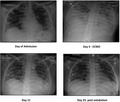

## 1 INTRODUCTION

The novel coronavirus disease 2019 (COVID‐19) has been declared as a “pandemic outbreak” and public health emergency of utmost international concern.^1^ With over 37 million confirmed cases and 1 million deaths (as of 12/10/2020), the pandemic continues to harm significant number of people worldwide. Approximately, 5% of the infected cases are complicated by hypoxia and respiratory failure.^2^ The reported prevalence of severe pneumonia among Chinese patients was as high as 5% with an estimated mortality rate of 2.3%‐3.83%.^3^, ^4^, ^5^ Partial immune suppression, physiological and anatomical changes, and multiple interaction with the healthcare system during pregnancy present an unprecedented challenge in managing this vulnerable population.^2^, ^6^, ^7^

Previous infectious outbreaks, such as H1N1 influenza virus, Zika virus, severe acute respiratory syndrome coronavirus (SARS‐CoV), and Middle East respiratory syndrome coronavirus (MERSCoV), have had significant adverse impact on maternal as well as perinatal outcomes.^6^, ^8^ Data collected from these patients demonstrated higher rates of intensive care unit admission, intubation, and death when compared with nonpregnant patients.^7^, ^9^ Current evidence demonstrates that 0.1%‐0.2% of all pregnancies are complicated by respiratory failure.^10^, ^11^

Despite promising outcomes, till date no pharmacological intervention has been proven effective to treat COVID‐19. Furthermore, no high‐quality evidence exists for the safety and efficacy of convalescent plasma in treating SARS‐COV2 infection. Inconclusive, limited clinical experience has been reported supporting the use Extracorporeal membrane oxygenation (ECMO) in the management of COVID‐19.^12^, ^13^ In the absence of any definitive therapy, the cornerstone of COVID‐19 treatment varies from symptomatic ambulatory care management to intensive care treatment.^14^

## 2 CASE HISTORY/EXAMINATION

We present a case of a 33‐year‐old pregnant woman at 32 weeks of gestation, who was referred to a tertiary care center following 8‐day history of malaise, cough, sore throat, and shortness of breath. Her past medical history revealed asthma (>10 years) on inhaled steroids and gestational diabetes. Prior to admission, she was taking vitamins and budesonide and reported no history of allergies to medications. She is a nonsmoker and no recent travel was reported.

During the current admission, her vital signs were as follows: temperature 37.4°C, heart rate—98 beats per minute, respiratory rate 25 br/min, blood pressure 98/63 mm Hg, and oxygen saturation (SpO_2_) 90% room air, 96% with nasal cannula. She complained of tiredness, lower backache while breathing, pertinent dry cough, and shortness of breath. No obstetric or fetal concerns were noted.

### 2.1 Diagnosis, investigation, and treatment

**FIGURE 1:**
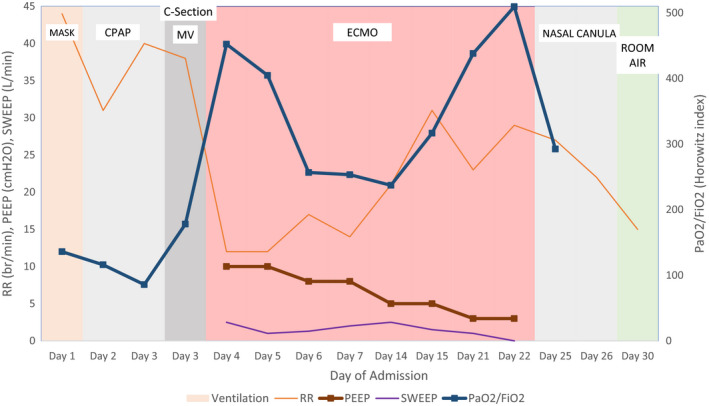
Ventilation flow sheet during hospitalization. (RR, respiratory rate (breaths/min); PEEP, positive end‐expiratory pressure (centimeters of water); SWEEP, ECMO gas flow (Liters/min); PaO_2_/FiO_2_, Horowitz index, partial pressure of oxygen arterial/fraction of inspired oxygen, ECMO, extracorporeal membrane oxygenation, MV, mechanical ventilation)

All relevant blood tests, nasopharyngeal swab/real‐time reverse transcriptase polymerase chain reaction (Rt‐PCR), and chest X‐ray were performed (Table 1). Table 1 highlights the variation in laboratory values during COVID‐19. Chest X‐ray demonstrated patchy ground glass pneumonic infiltrates in both lung fields suggesting clinical correlation for pneumonia (Figure 1). Nasopharyngeal and throat swabs confirmed positive for SARS‐CoV‐2 infection.

**TABLE 1:** Laboratory values (maternal) for the first 10 d of admission (all underlined, deviates from the normal range)

Lab values	Reference range	D1	D2	D3	D3	D5	D6	D7	D8	D9	D10
Hemoglobin (g/dL)	12‐15	11.6	11.2	12.8	10.6	11.5	8.1	8	9.1	9.1	8.9
WBC (10^3^/uL)	4‐10	5.08	6.2	8.1	19.2	11.9	11.0	9.3	9.5	8.8	8.6
Lymphocyte (10^3^/uL)	1‐3	0.6	0.4	0.5	0.4	0.5	0.6	0.5	0.3	0.4	0.1
Platelets (10^3^/UL)	150‐400	126	153	189	257	291	258	308	348	379	329
C‐reactive protein(mg/L)	0‐5	168	149	170	66	103.9	90.5	NA	22.2	NA	7.8
Procalcitonin (micrograms/L)	<0.5	NA	1	0.42	0.29	0.18	0.16	0.11	0.06	0.06	0.05
Creatinine (mg/dL)	44‐80	35	25	29	32	60	67	53	49	39	40
Aspartate transaminase (units/L)	0‐32	36	39	43	32	68	124	163	204	232	216
Alanine transaminase (units/L)	0‐33	16	17	19	16	35	76	136	205	332	424
Alkaline phosphatase (units/L)	35‐104	132	158	192	155	158	97	90	85	86	80
Prothrombin time (sec)	9.7‐11.8	9.9	NA	NA	9.2	9.6	10.1	9.9	10.9	11.4	11.9
Glucose mmol/L	3.3‐5.5	7.8	5.9	6.7	6.9	11.8	9.5	7.5	4.4	7.2	7.9
Ferritin (micrograms/L)	12‐160	236	253	NA	355	NA	NA	1039	1110	1431	1530
Lactate dehydrogenase (units/L)	135‐214	NA	NA	NA	386	NA	NA	NA	868	NA	848
Fibrin D‐dimers (ng/mL)	0‐0.44	2.01	NA	NA	1.01	3.8	NA	2.7	2.1	NA	3.1

Following positive Rt‐PCR, on day 1 of the hospital stay the patient was commenced on HMC's COVID‐19 treatment protocol, that is, chloroquine 400 mg once daily for 10 days, azithromycin 500 mg once daily for 10 days, oseltamivir 150 mg twice orally for 10 days, and intravenous ceftriaxone 2 gm once daily for 10 days. Within few hours, the patient developed severe hypoxemia and acute respiratory distress (RR: 35 b/min) and was unable to complete sentences.

The patient was transferred to a negative pressure intensive care room with multidisciplinary expertise to manage critically ill obstetric patients. Additional oxygen support was provided, nonbreather mask 15 L/mins. As patient's condition continued to worsen, methylprednisolone 40 mg was administered to decrease the inflammatory responses in the lungs. On day 2, the patient was started on noninvasive ventilation; however, she complained of uneasiness, shortness of breath, and pain while breathing. As a part of the treatment, protocol Kaletra^®^ lopinavir/ritonavir (80/20 mg) and one dose of tocilizumab 400 mg (for hyperinflammation caused by cytokine release) were administered (informed consent was obtained). On day 3, as the respiratory functions deteriorated, a multidisciplinary team, including internists, medical team, obstetric specialty, anesthetist decided to intubate her.

As the patient required deep sedation to tolerate lung‐protective ventilation, continuous sedation/analgesia with propofol: 50 mcg/kg/min + fentanyl: 5 mcg/kg/hr was administered aiming the Richmond Agitation and Sedation Scale (RASS) ^15^ more than −4. Despite intubation and other medical interventions, the respiratory functions and static compliance worsened remarkably, and positive end‐expiratory pressure (PEEP) and Fi02 were kept high.

The cardiotocography demonstrated no fetal heart acceleration and nonreassuring fetal outcomes anticipated mostly due to maternal hypoxemia. Considering persistent deterioration in maternal respiratory function and signs of fetal distress, it was decided to terminate the pregnancy by cesarean section (C‐section). Magnesium sulfate 2 gm infusion was commenced for fetal neuroprotection and was monitored for toxicity. The patient underwent an uncomplicated lower segment transverse cesarean section delivering a 1900 gms baby boy, with a blood loss of 300 mL. As per current recommendations, there was no need for a delayed cord clamping, and the baby was separated from the patient immediately.

On day 4, the patient encountered prolonged QTC intervals anticipated mostly due to hydroxychloroquine/+Kaletra^®^ lopinavir/ritonavir. Hydroxychloroquine was withheld, and timely monitoring was recommended. The patient encountered life‐threatening hypoxia and severe acute respiratory failure postoperatively. ECMO team was consulted, and veno‐venous ECMO bifemoral cannulation was initiated after consent was obtained from the husband. On day 5, during infectious disease consultation ribavirin was added and 2 units of convalescent plasma were administered to improve her respiratory mechanisms. On day 6, the ECMO team decided to stop cisatracurium and noradrenaline and to wean off sweep gas aiming for oxygen saturation >88% and PH >7.25. Cabergoline 1 mg, potent dopamine receptor agonist (to inhibit milk production) was administered to avoid breast engorgement and pain.

**FIGURE 2:**
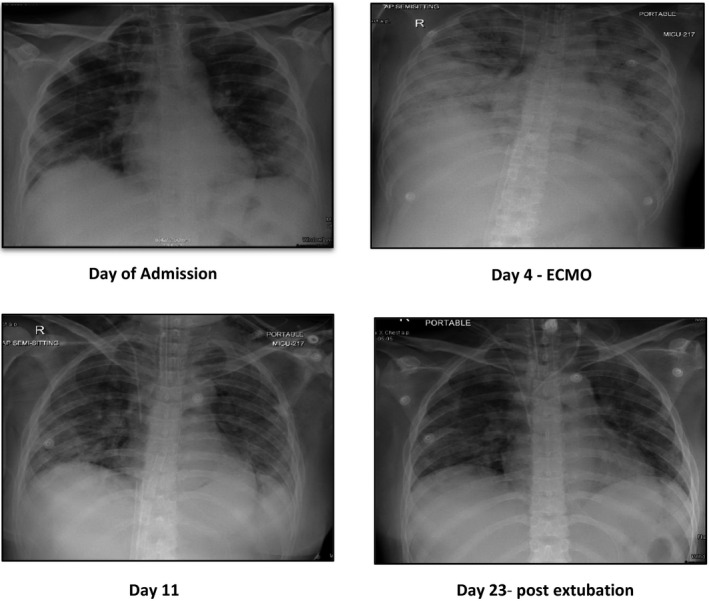
Chest X‐ray demonstrating ARDS

On day 14, on ECMO, the liver enzymes were elevated, due to antiviral medications, following which the ribavirin was withheld. The inflammatory markers drastically reduced following administration of methylprednisolone 20 mg. On day 18, the patient developed hematuria and hematoma at the C‐section wound. Therefore, the antibiotics were started and low dose of fibrinogen was replaced. On day 19, the patient developed subcutaneous emphysema due to Gram‐positive bacteria, which was treated with cefepime, later changed to piperacillin tazobactam sodium 4500 mg. On day 23, following an improved clinical and respiratory functions, ECMO decannulation was performed and was well tolerated. After 24 hrs, the patient was extubated on high flow nasal oxygen (2L), echocardiogram revealed no evidence of infective endocarditis. On day 25, following improved clinical findings, the patient was shifted from the intensive care unit to a step‐down unit with continuous monitoring. The patient was clinically stable and was discharged 2 weeks later Figure 2.

### 2.2 Neonatal summary

Following a preterm delivery, the neonatal APGAR scores documented at 1, 5, and 10 minutes were 2, 3, and 7, respectively, pH at birth 7.0203, Sarnat stage grade 1. However, an hour after delivery, the newborn developed severe asphyxia and was transferred to neonatal intensive care unit for resuscitation and intubation. The baby received one dose of surfactant and was extubated after 16 hours to nasal continuous positive airway pressure. The newborn was tested twice (14 days interval) for COVID‐19 IgG and IgM and was found negative in both the occasions. The baby was discharged on day 14 on stable vitals.

## 3 DISCUSSION

We report a multidisciplinary approach to treating and complete recovery of acute respiratory failure and severe pneumonia secondary to SARS‐COV2 infection during pregnancy. A plethora of studies have demonstrated the management of mild–moderate cases of SARS‐COV2 infection in pregnancy with positive outcomes.^2^, ^10^, ^16^ However, very few studies have reported the management of critically ill patients, particularly in pregnancy. Pneumonia during pregnancy is often accompanied by hospitalization and critical care management including ventilatory support.^20^ Although the treatment of pneumonia during pregnancy mirrors that of nonpregnant state, the use of convalescent plasma and ECMO in pregnancy is rare.^9^, ^11^, ^17^

The clinical presentations, symptoms, and the radiological findings in our case were consistent to previous case reports,^3^, ^18^, ^19^, ^20^ of SARS‐COV2 infection. A nationwide population‐based cohort (n = 1942) reported pregnant women with viral pneumonia (other than COVID‐19) demonstrated higher risk of preterm birth, intrauterine growth retardation low birthweight, and poor Apgar scores when compared to those without pneumonia.^21^ Hence, as demonstrated in this case, an early delivery is considered as an alternative for critically ill pregnant women with acute respiratory distress syndrome (ARDS).

In terms of therapeutic management, no specific pharmacological agent or vaccine to treat COVID‐19 is available.^12^ Once COVID‐19 was confirmed, hydroxychloroquine, azithromycin, oseltamivir, intravenous ceftriaxone, and methylprednisolone were administered and are considered safe in pregnancy and have been used extensively to treat COVID‐19.^22^ Furthermore, current evidence on safety and efficacy of hydroxychloroquine for the treatment of COVID‐19 reveals the drug was not associated with difference in overall mortality and limited evidence has reported more adverse events than standard care.^22^ However, there is a paucity of evidence regarding the use of antimalarial and antiviral therapy in treating SARS‐COV2 infections in pregnancy.^22^, ^23^ Therefore, even in this case, it is unclear whether the empirical use of these medications had any role in the recovery of our patient. Tocilizumab (monoclonal antibody IL‐6 receptor antagonist) was administered postoperatively, due to the deteriorating respiratory functions, hemodynamic instability, and persistently elevated inflammatory markers. Several COVID‐19 studies have demonstrated improved respiratory functions, and successful recovery in patients receiving one dose.^23^, ^24^, ^25^

Anecdotal evidence from previous viral infections including Ebola, SARS‐CoV, H5N1 avian influenza, and H1N1 influenza suggests the use of convalescent plasma containing neutralizing antibody is effective.^26^, ^27^, ^28^, ^29^ Food and Drug Administration has conditionally approved (under special circumstances) the use of convalescent plasma to treat critically ill COVID‐19 patients.^30^ A meta‐analysis which investigated the effectiveness of convalescent plasma in SARS coronavirus infection and severe influenza reported significant reduction in viral loads and mortality.^31^ In this case, the transfusion of convalescent plasma demonstrated improved clinical outcomes, and COVID‐19‐specific inflammatory markers were significantly improved. More recent studies have also advocated the use of prophylactic‐dose anticoagulation in all COVID‐19 patients admitted to the hospital or ICUs unless contraindicated.^32^

There is a scarcity of evidence behind the use of lung‐protective ventilation and ECMO in COVID‐19 infection during pregnancy.^10^ The use of ECMO in pregnancy and postpartum is rare. An estimated 40% of pregnant or postpartum women admitted to ICU are complicated by ARDS or cardiac arrest.^33^, ^34^ Previous reports which demonstrated improved maternal survival,^35^, ^36^ the use of VV‐ECMO in this patient is expected to have potentially resulted in positive respiratory outcomes and successful recovery. Furthermore, providing adequate rest to lungs using VV‐ECMO was necessary to avoid ventilator‐associated and oxygen‐induced lung injury. Although the current case predominantly focuses on treating the respiratory symptoms, atypical manifestations such as altered mental status, dizziness, rashes, and blood clots should also be considered equally important while treating COVID‐19.^37^

## 4 CONCLUSION

Through this case, we demonstrate the importance of involving multidisciplinary team in decision making, as managing maternal complications as well as reassuring fetal well‐being during such critical period is challenging. We have thus confirmed the feasibility of using convalescent plasma and ECMO during early postnatal period in critically ill obstetric patients with respiratory failure. The use of tocilizumab, convalescent plasma, followed by intensive care management with intubation and ECMO, might have potentially contributed to the complete recovery of the mother and the newborn. Whether antimalarials‐hydroxychloroquine (in particular) and/or antivirals are effective in treating COVID‐19 remains unknown.

## EXPERTISE

Our team comprises of experts from obstetrics specialty, critical care medicine, emergency medicine, infectious disease, clinical pharmacy, health policy, and research. We have worked together previously with a proven track record of researching in around obstetric and perinatal population.

## CONFLICT OF INTEREST

The authors declare that there is no conflict of interest.

## AUTHOR CONTRIBUTIONS

All authors have made substantial contribution to the case report. SY: (consultant obstetrician) and MH: (pharmacy executive director) have contributed to the conceptualization of the case report and were responsible for supervision, planning, and execution. SH: is a senior consultant and head of obstetric emergency who was involved in the initial assessment of the case. ZM: is the primary obstetrician (senior consultant) who was involved in treating the case along with TR & IB: (obstetric consultant) and MJ: (specialist obstetrician). AA: is a senior research fellow and ECMO consultant who managed the case in intensive care unit. AM: is a senior consultant at the emergency department, involved in the initial management of the case. MM: is infectious disease consultant and provided valuable input to the manuscript and managing the COVID‐19 and related complications. AT and FM: have contributed by interpreting the laboratory and radiological findings. HS: is consultant neonatologist who managed the newborn in the NICU. PR and WK: are pharmacy administrative, involved in the acquisition of the financial support, scientific review, and verification for the validity of content. BT: is a clinical pharmacy specialist and doctoral researcher who took the lead in writing the manuscript in consultation with SY, MH, AM, PR, FM. All authors discussed, reviewed, and edited the case report. All authors agreed to the final version prior to its submission.

## ETHICAL APPROVAL

Ethical approval to report this case was obtained from the institutional review board at Hamad Medical Corporation.

## INFORMED CONSENT

Written informed consent was obtained from the patient for their anonymized information to be published in this article and shall be presented on request.

## CASE REGISTRATION

This case has been registered as a preprint at Authorea.^38^

https://doi.org/10.21203/rs.3.rs‐36328/v1

This case has been presented in accordance with CARE case report guidelines.

## Data Availability

The data related to this case study are not publicly available as it may compromise the privacy of the patient and the participants. However, the data that support the findings of this study are available from the corresponding author, upon reasonable request.
